# Identification of hydatidosis-related modules and key regulatory genes

**DOI:** 10.7717/peerj.9280

**Published:** 2020-06-18

**Authors:** Jijun Song, Mingxin Song

**Affiliations:** 1 College of Veterinary Medicine, Northeast Agricultural University, Harbin, Heilongjiang, China; 2 Harbin Weike Biotechnology Co., Ltd., Harbin Veterinary Research Institute, CAAS, Harbin, Heilongjiang, China; 3 Heilongjiang Key Laboratory for Zoonosis, Harbin, China

**Keywords:** Echinococcosis, Host, Module, miRNA-184

## Abstract

**Background:**

*Echinococcosis* caused by larval of *Echinococcus* is prevalent all over the world. Although clinical experience showed that the presence of tapeworms could not be found in liver lesions, the repeated infection and aggravation of lesions still occur in the host. Here, this study constructed a multifactor-driven disease-related dysfunction network to explore the potential molecular pathogenesis mechanism in different hosts after *E.multilocularis* infection.

**Method:**

First, iTRAQ sequencing was performed on human liver infected with *E.multilocularis*. Second, obtained microRNAs(miRNAs) expression profiles of humans and canine infected with *Echinococcus* from the GEO database. In addition, we also performed differential expression analysis, protein interaction network analysis, enrichment analysis, and crosstalk analysis to obtain genes and modules related to *E.multilocularis* infection. Pivot analysis is used to calculate the potential regulatory effects of multiple factors on the module and identify related non-coding RNAs(ncRNAs) and transcription factors(TFs). Finally, we screened the target genes of miRNAs of *Echinococcus* to further explore its infection mechanism.

**Results:**

A total of 267 differentially expressed proteins from humans and 3,635 differentially expressed genes from canine were obtained. They participated in 16 human-related dysfunction modules and five canine-related dysfunction modules, respectively. Both human and canine dysfunction modules are significantly involved in BMP signaling pathway and TGF-beta signaling pathway. In addition, pivot analysis found that 1,129 ncRNAs and 110 TFs significantly regulated human dysfunction modules, 158 ncRNAs and nine TFs significantly regulated canine dysfunction modules. Surprisingly, the *Echinococcus* miR-184 plays a role in the pathogenicity regulation by targeting nine TFs and one ncRNA in humans. Similarly, miR-184 can also cause physiological dysfunction by regulating two transcription factors in canine.

**Conclusion:**

The results show that the miRNA-184 of *Echinococcus* can regulate the pathogenic process through various biological functions and pathways. The results laid a solid theoretical foundation for biologists to further explore the pathogenic mechanism of *Echinococcosis*.

## Introduction

Echinococcosi*s*, also known as hydatidosis, is a worldwide chronic zoonosis caused by larval of *Echinococcus multilocularis* (*E.multilocularis*) that mainly affects humans, livestock and wild mammals (Maldonado et al., 2017; Zhang et al., 2019). *E.multilocularis* is mainly distributed in holarctic regions, including Asia, North America, and Europe (Oksanen et al., 2016). In China, there are many Echinococcosis-prone areas, such as Qinghai, Gansu, and Sichuan (Massolo et al., 2014). The life-cycle of *E.multilocularis* involves small rodent intermediate hosts such as arvicolids and wild or domestic canid definitive hosts such as the foxes, wolves, dogs, and cats. The host is infected by accidentally eating the eggs of *E.multilocularis*, causing tumor-like lesions of the liver (Li et al., 2018b). Human can act as abnormal intermediate hosts and become infected when ingesting eggs released into vegetables and food by adult worms. Then, the parasite larvae travel to internal organs, mainly the liver (Conraths et al., 2017). Hydatidosis not only affects the world economy, but also seriously endangers the international public health problem (Paoletti et al., 2019). Echinococcosis has been reported as a prioritized neglected disease by the World Health Organisation (WHO) in 2012 (Maldonado et al., 2017). In 2015, according to WHO estimates, Echinococcosis was the cause of 19,300 deaths and approximately 871,000 disability-adjusted life years (DALYs) worldwide each year, and Echinococcosis-related treatment costs and livestock industry losses estimated at 3 billion dollars annually.

Therefore, it is urgent to study the pathogenesis and treatment of hydatidosis. Fortunately, many biologists and medical researchers have devoted themselves to the exploration of hydatidosis-related diseases and achieved great results. Research by Li et al. (2018a) shows that *E.multilocularis* is contagious in two stages: one is egg, the only stage for primarily infecting humans and intermediate host; and another stage is protoscolex, the only stage for infecting the definite hosts of the worms. Previous studies have shown that extracellular vesicles (EV) secreted by *E.multilocularis* participate in host-parasite interactions by exchanging biomolecules, and then play a role in the process of infection (Zheng et al., 2017). In addition, ECT 53, a homologous gene of p53, was found to play an important role in the regulation of antioxidant stress and apoptosis induced by oxidative stress in *E.multilocularis (Cheng et al., 2015)*. EmSOX 2, as a member of Sox transcription factor family, is actively expressed in germinating cells of *E.multilocularis* proliferation, and is a potential regulator of *E.multilocularis* germinating cells (Cheng et al., 2017a).

miRNA play an important role in the regulation of parasite infection. Surprisingly, there is a significant correlation between the expression level of miRNA and their corresponding target genes, mainly involved in *E.multilocularis* infection and regulation of cytokine activity and immune response (Guo & Zheng, 2017). Zheng (2018) confirmed through animal experiments that downregulated miR-222-3p is capable of modulating macrophage immune functions, possibly contributing to the pathogenesis during *E.multilocularis* infection. Similarly, specific amplification of two kinds of miRNAs (emu-miR-10 and emu-miR-227) derived from parasites has been found in previous reports that they contribute to the role of these two miRNAs as diagnostic targets for hydatidosis (Guo & Zheng, 2017). In this study, we found that miR-184 plays a regulatory role in both human and canine modules. Several targets for miR-184 have been described, including that of mediators of neurological development, apoptosis and it has been suggested that miR-184 plays an essential role in development (Li et al., 2011). Liu et al., (2010) showed that Methyl-CpG binding protein 1 (MBD1) regulates the expression miR-184. High levels of miR-184 promotes cell proliferation, whereas inhibition of miR-184 rescued phenotypes associated with MBD1 deficiency. We speculate that miR-184 played an important role in hydatidosis.

Some signaling pathways and key pathway factors are also involved in the infection of *Echinococcus*. EGFR/ERK signaling pathway can stimulate the growth and development of larvae by promoting the proliferation of germinating cells in host (Cheng et al., 2017b). Interestingly, the granular layer of *E.granulosus* has anti-inflammatory effects by inhibiting PI3K/Akt activation and growth factor-driven Akt phosphorylation and macrophage proliferation (Seoane et al., 2016). In addition, host insulin can stimulate the development of parasites in the liver (Hemer et al., 2014). Up to now, only benzimidazole, albendazole and tolbendazole have been allowed to be used in the treatment of human hydatidosis (Siles-Lucas et al., 2018). Previous clinical experience has shown that *E.multilocularis* can cause repeated infection of the host, and the clinical and pathological symptoms are gradually aggravated. Therefore, it is imperative to comprehensively explore the basic detailed mechanism and key regulatory molecules of *E.multilocularis* infection.

Here, we explore the potential pathogenic mechanism of *E.multilocularis* through in-depth analysis of the sequencing data of human and canine *E.multilocularis* infection. The comprehensive strategy based on functional modules provides rich resources and theoretical guidance for biologists to further explore its therapeutic mechanism.

## Material and Methods

### Blood and tissue samples

On February 8, 2019, we obtained liver tissues from five patients and divided each patient’s liver tissue into the lesion area, marginal area and distal area. Liver tissues from patients surgery were instantly immersed in liquid nitrogen and preserved at −80  °C. Next, we obtained the plasma of 5 patients with hydatidosis and the plasma of 5 healthy people (blood drawn during the physical examination) as controls. Samples of patients with hydatidosis were taken from Heilongjiang Infectious Diseases Hospital. All samples were confirmed by experienced pathologists. Written informed consent was obtained from all patients. The Medical Ethics Committee of Heilongjiang Provincial Hospital for the Prevention and Treatment of Infectious Diseases approved the study of human liver infected with E. coli and the study of GEO data (No. 2017(KY-E-89356)). All canine samples were taken from which infected with *Echinococcus* multilocularis in the College of Veterinary Medicine, Northeast Agricultural University. Blood was obtained from three dogs infected with *Echinococcus* on February 13, 2019, and blood from three normal dogs was used as a control. Human or canine samples were collected according to the International Ethical Guidelines for Biomedical Research involving Human and Subjects. The Northeast Agricultural University Animal Care and Welfare Committee granted ethical approval to carry out the study within its facilities (201707056).

### qPCR

According to the manufacturer’s instructions, TRIzol (Invitrogen) and miRNeasy mini kit (QIAGEN) were used to isolate total RNA from liver tissue, which can effectively recover all RNA species including miRNA. RNA was transcribed into cDNA using a reverse transcription kit. qPCR reaction was carried out using the miRcute Plus miRNA qPCR Detection Kit. The qPCR program begins the initial 3 min denaturation step at 95 °C to activate the hot-start iTaqTM DNA polymerase. This was followed by 45 cycles of denaturation at 95 °C for 10 s and annealing and extension at 60 °C for 45 s. The internal reference genes are beta-actin and U6.

### Preparation and Quantification of Protein

Tissue samples were placed in a pre-cooled abrasive mortar and manually ground under liquid nitrogen until nitrogen evaporated. Tissue proteins were extracted by Kit Method and operated according to manufacturer’s instructions (Solarbio). In addition, bovine serum albumin (BSA) was used as the standard to determine protein concentration by Bradford method. Use the Quick Start Bradford Protein Assay Kit (Bio-Rad Laboratories) according to the manufacturer’s instructions.

### iTRAQ labeling

The samples of each group were taken about 100 ug, and the proteins were enzymatically hydrolyzed and labeled according to the instructions of iTRAQ Reagent-4plex Multiplex Kit.

### Identification of Peptides by LC-MS/MS

The labeled peptide was loaded twice on a nano-RP column with Dionex ultimate 3,000 nano-HPLC system (Shimadzu), and then eluted by ACN gradient. The eluent was injected directly into the Q-Exactive mass spectrometer (Thermo Fisher Science) and operated in a positive ion mode with a full MS scan of 350 to 2,000 m/z. The peak area ratio of iTRAQ reported ions reflects the relative abundance of peptide segments. The ratio of protein expression was calculated based on the peak area ratio of the same protein. The LC-MS/MS steps were repeated twice on the same group of samples.

### Data resources

The gene expression data was downloaded from the NCBI Gene Expression Omnibus (GEO) database. The GSE105098 dataset includes four normal dogs, four dogs infected with *E.multilocularis* for the first time, four dogs repeatedly infected with this parasite four times (Kouguchi et al., 2018). Small intestine from dogs was used for total RNA extraction. In each group, equal amount of purified total RNAs from 4 dogs were pooled and then used for microarray analysis. The chip platform is Agilent-021193 Canine (V2) Gene Expression Microarray. The GSE64705 dataset contains 6 samples (Macchiaroli et al., 2015). Small RNA libraries from protoscoleces and cyst walls of *E.canadensis* G7 and protoscoleces of *E.granulosus* sensu stricto G1 were sequenced using Illumina technology. For each sample type, two libraries were constructed from two independent samples in order to have biological replicates.

### Differential expression analysis

In this study, differential expression analysis was performed by R language limma package (Ritchie et al., 2015). We first used background correct function for background correction and standardization. Then, based on the quantile normalization method of normalize Between Array function, the control probes and low-expression probes were filtered for high-quality standardized data. The lmFit and eBayes functions of limma package with default parameters were used to identify differentially expressed genes with *p* value > 0.01.

### Mining hydatidosis-related functional modules

Firstly, we searched interactors (score > 950) of differentially expressed genes in humans and canine from the STRING database (Franceschini et al., 2013) to construct the protein-protein interaction network, respectively. Then, ClusterONE, a plugin in Cytoscape, was applied to identify hydatidosis-related functional modules with default parameters (Nepusz, Yu & Paccanaro, 2012). ClusterONE is a method for detecting potentially overlapping protein complexes from protein-protein interaction data. The PPI data can be represented as undirected graphs, in which nodes represent proteins and edges represent interactions between pairs of proteins. The reliability of such effects can be estimated and included as edge markers (weights).

### GO function and KEGG pathway enrichment analysis

Exploring the function and signal pathway of genes is often an effective means to study the molecular mechanism of diseases. Therefore, the enrichment analysis was performed on all module genes with R language Cluster profile package (Go function: *p* value Cutoff = 0.01, *q* value Cutoff = 0.01; KEGG pathway: *p* value Cutoff = 0.05, *q* value Cutoff = 0.2).

### Analysis of crosstalking relationships among modules

First, Python was used to extract the original PPI network (score> 950) from the STRING database to generate 1000 random networks without changing the size of the network and the degree of each node. Then, the number of interaction pairs between two modules was calculated in each random network and the original network. Finally, the number of interaction pairs in original network was compared with the number of interaction pairs under random background. If the number of interaction pairs between modules in original network was significantly larger than the number of interaction pairs in a random context, the module pair was called crosstalk (Hopf et al., 2019). The method of calculating significant crosstalk is as follows: under the background of random network, the number of interaction pairs between modules in N random networks is larger than that in real networks, and is counted as n. The formula for calculating *p* value is: *p* = *n*∕*N* (in this work, *N* = 1000). When *p* < 0.05, it means there is crosstalk interaction between modules.

### Identifying the key genes in modules

According to the interaction relationships in each module, the connectivity of each module gene was calculated using Cytoscape software. Higher connectivity indicates that the gene has an important role in this module. The most connected genes in each module are considered key genes.

### Exploring TFs and ncRNAs significantly regulated on modules

In order to further explore the regulation of hydatidos related modules, we downloaded ncRNA-microRNA (protein) interaction pairs with score ≧0.5 from RAID v2.0 database and all human transcription factor target data from TRRUST v2.0 database as background set for pivot analysis (Yi et al., 2017). Pivot analysis refers to finding a regulator with at least two interactions with module in target pairs and with the significance of the interaction between the regulator and the module based on hypergeometric test (*p* value < 0.01).

## Result

## Dysfunction of gene expression in hydatidosis

**Figure 1:**
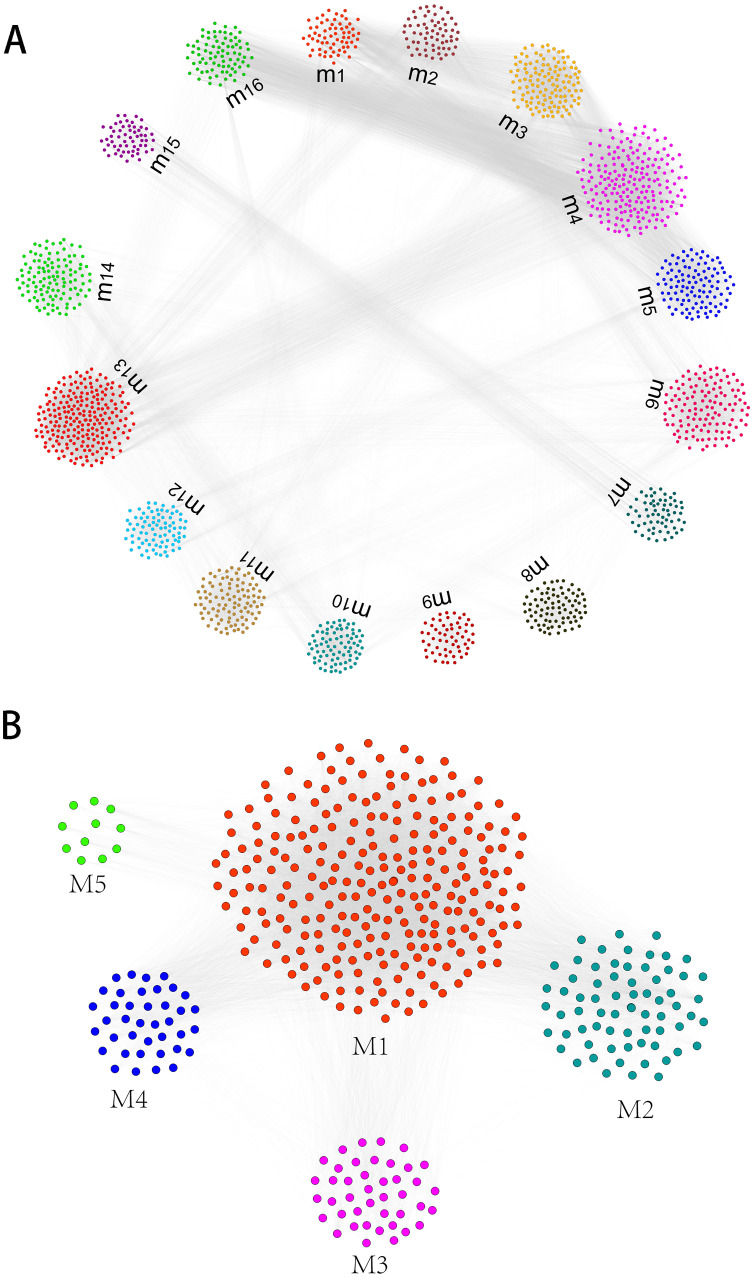
The identification of functional module networks. (A) Based on human interaction proteins, the protein interaction network map of 1,623 protein was obtained, and the genes were clustered into 16 functional module networks (m1 to m16). Each color represents a module, and gray lines represent connections between protein. (B) Based on canine interaction proteins, 470 protein interaction network maps were obtained and clustered into 5 functional module networks (M1 to M5). Each color represents a module, and gray lines represent connections between protein.

Gene expression profiles of humans and canine infected with *E.multilocularis* were obtained. Compared with the control group, we identified a total of 1,937 differentially expressed genes in the first-infection group canine, of which 1,400 were up-regulated and 537 were down-regulated. Compared with the control group, there are 1,698 differentially expressed genes in the repeated-infection group canine, of which 1,274 are up-regulated and 424 are down-regulated (Table S1). A total of 267 differentially expressed proteins, 101 up-regulated and 166 down-regulated were identified in human hepatic lesions and margins samples compared to control samples (Table S2). We suggest that these differentially expressed genes may be associated with hydatidosis. Then, for different host, we constructed protein-protein interaction (PPI) networks and identified hydatidosis-related modules in PPI network, respectively. Among them, in humans infected with *E.multilocularis*, a total of 16 modules were identified, namely m1 to m16 (Fig. 1A). In canine infected with *E.multilocularis*, a total of 5 modules were identified, namely M1 to M5 (Fig. 1B). These modules may participate in different functions and pathways, representing different post-infection regulatory mechanisms of echinococcosis.

## Identification of dysfunction modules through function and pathway

In order to further understand the biological characteristics of the two module sets, we performed GO function and KEGG pathway enrichment analysis, respectively. We have collected a wealth of GO terms (Table S3). In GO terms, the human module was significantly enriched in 2,510 cell compositions, 3,071 molecular functions, and 23,185 biological processes. Canine modules were involved in 504 cell components, 866 molecular functional and 5,393 biological processes. Based on functional analysis, we observed that these modules tend to enrich a variety of disease-related functions, such as response to “endoplasmic reticulum stress” and response to “transforming growth factor beta”. KEGG enrichment analysis shows that human modular genes participate in 264 functional pathways and canine modular genes participate in 240 functional pathways (Table S4). Human modular genes are significantly enriched in PI3K-Akt signaling pathway and TNF signaling pathway, which are significantly related to the inflammatory reaction in the occurrence and development of hydatidosis in human. Canine modular genes were mainly involved in signal pathways such as MAPK signaling pathway and p53 signaling pathway. These pathways play important role in the apoptosis process of canine *E.multilocularis*. In view of the close relationship between the function and pathway of module genes and the progression of hydatidosis, we identified these two modules as dysfunction modules.

## Crosstalk between modules and key genes in modules

**Figure 2:**
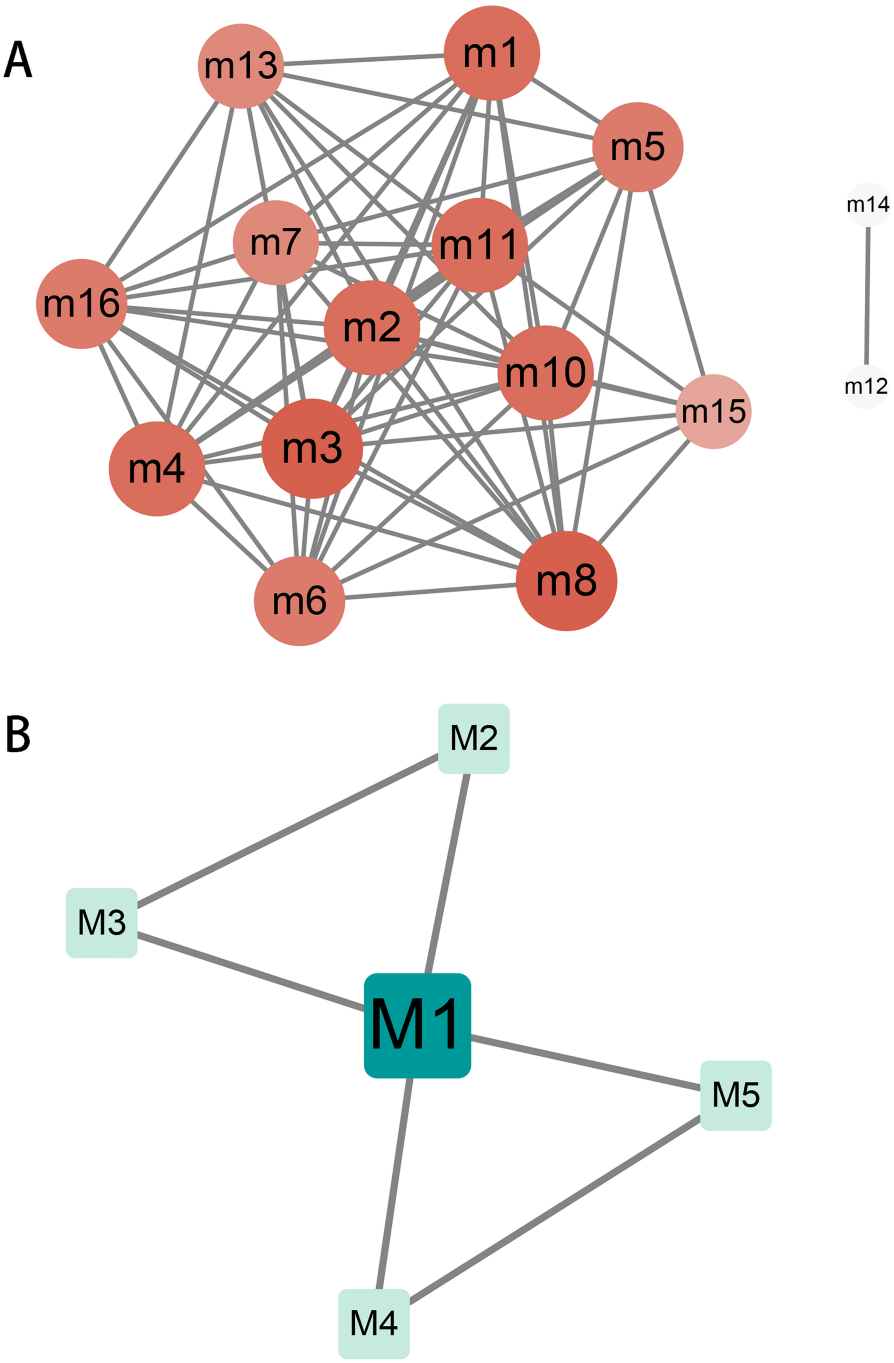
The crosstalk analysis of modules of humans or canine. (A) There are 68 significant crosstalk (module crosstalk interaction pairs) between human hydatidosis modules. (B) Canine echinococcosis showed that five modules had interaction. The darker the color, the stronger the module crosstalk. The gray lines represent the crosstalks between modules.

The relationship and regulation between genes have always been complex and changeable. Exploring the complex interaction between modules will help us to understand the molecular mechanism during the progression of hydatidosis. Therefore, we performed crosstalk analysis between modules based on the interaction relationships between module genes. We identified 60 significant crosstalk relationships among human dysfunction modules (Fig. 2A), and five relationships among canine dysfunction modules (Fig. 2B). The crosstalks may represent the relationship between modules, which together regulate the progression of hydatidosis. In addition, the role of important genes in modules was more crucial. Therefore, based on the degree distribution of genes in modules, the genes with the highest connectivity in each module are considered as key genes. Then, we analyzed 16 human modules and found that key genes in 12 modules played a regulatory role in the occurrence and development of hydatidosis. In addition, in 5 canine-related modules, key genes related to hydatidosis were identified (Table S5). It is worth noting that among all the key genes of patients with hydatidosis, the connectivity of RPS5 is 148, which is the most connected endogenous gene. In canine, EHMT1 with a connectivity of 73 is considered the most critical endogenous gene.

## The regulation of miR-184 mediated by ncRNAs/TFs in the progression of hydatidosis

**Figure 3:**
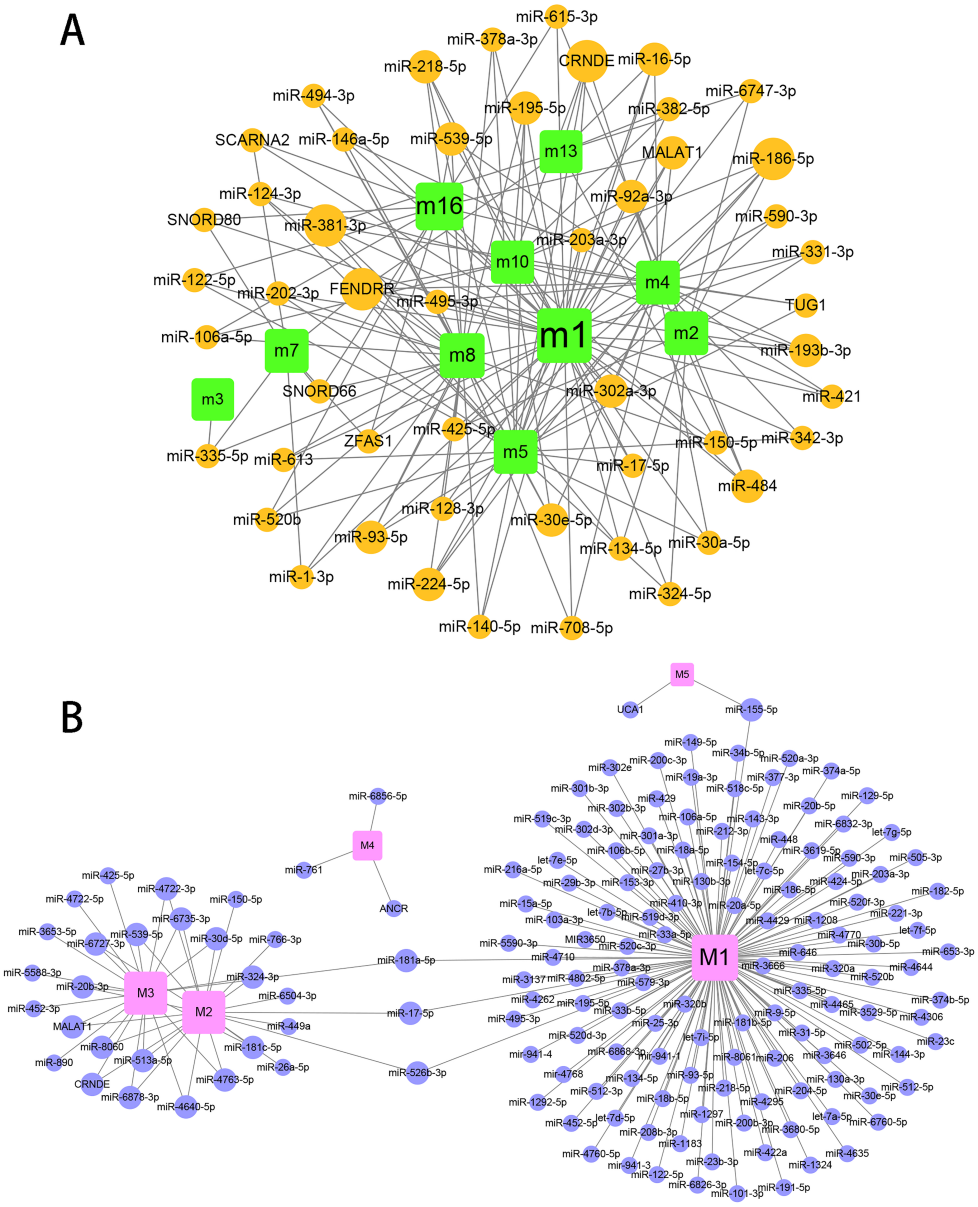
Prediction results for ncRNAs that regulate the module. (A) ncRNA-pivot regulates human modules. Each circle represents a ncRNA, and each square represents a module. (B) ncRNA-pivot regulates canine modules. The larger the node, the stronger the regulatory role. Each circle represents a ncRNA, and each square represents a module (M1 to M5).

**Figure 4:**
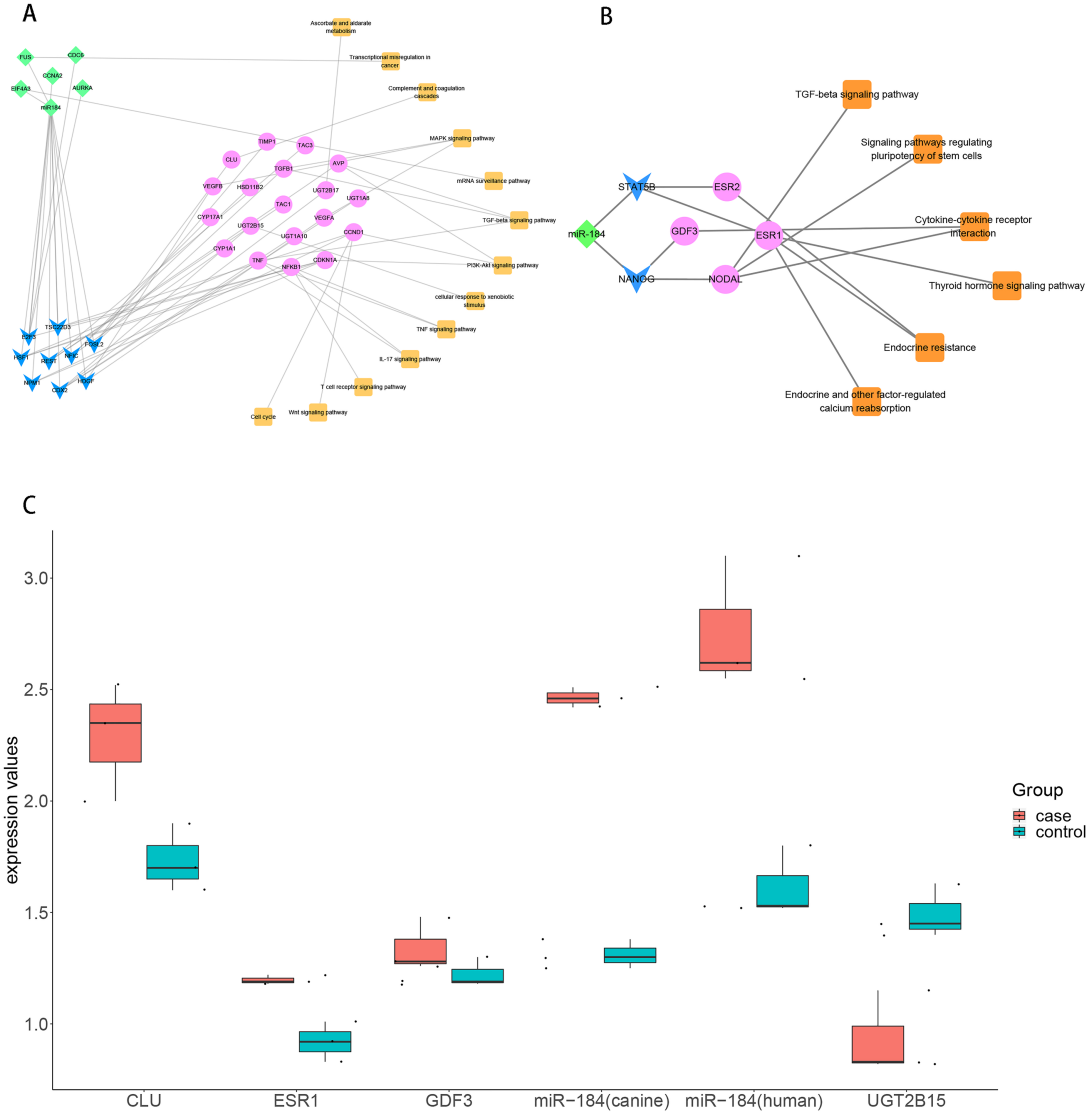
Analysis and verification results of key TF. (A) MiR-184 regulates human module gene through TF and ncRNA, then participates in hydatidosis-related signaling pathways. The diamond represents ncRNA, the arrow represents TF, the circle represents target gene, and the square represents pathway. (B) MiR-184 regulates canine modules gene through TF, then participates in hydatidosis-related signaling pathways. The diamond represents ncRNA, the arrow represents TF, the circle represents target gene, and the square represents pathway. (C) Box plot showing qPCR validation of key genes. Red represents case group, blue represents control group.

To explore the role of ncRNA during the progression of hydatidosis, pivot analysis was performed to identify ncRNAs, which significantly regulated modules. In total, we identified 158 ncRNAs that significantly regulate pathogenic module in canine *E.multilocularis* infection and 1,129 ncRNAs that significantly regulate pathogenic module in human *E.multilocularis* infection (Table S6, Figs. 3A) and  3B. Especially, miR-184 has homologues in *E.multilocularis*. SLC38A3, a target of miR-184, is homologous in human and canine. Therefore, we infer that miR-184 aggravates the pathological changes of human infected by regulating SLC38A3. It can be concluded that miR-184 plays an important role in the pathogenesis of hydatidosis. The pathogenic process of hydatidosis is also closely related to the disorder of TFs. Similar to ncRNAs, we performed pivot analysis to predict the regulation relationships between TFs and modules (Table S7). The results showed that 110 TFs had significant transcriptional regulation effects on the dysfunction module of *E.multilocularis* infection in humans. Statistical analysis showed that NFKB1 significantly regulated 6 dysfunction modules, which may participate in the inflammatory process and promote physiological disorders in the process of *E.multilocularis* infection. RELA and SP1 also play an indispensable role in the process. We also found that 9 TFs regulate the dysfunction of canine *E.multilocularis* infection. TP53 regulated one dysfunction module and plays a central role in the response to oxidative stress and DNA damage. In the regulatory network, miR-184 regulates target genes through 9 TFs and participates in 13 KEGG pathways (Fig. 4A). Among the TFs, FOSL2 may up-regulates CLU of module 11 and regulates inflammatory response. CDX2 could down-regulates UGT2B15 of module 12, and participates in cellular response to xenobiotic stimulus and other functions. In the canine regulatory network, miR-184 regulates 4 target genes through 2 TFs and participates in 6 KEGG pathways (Fig. 4B). Similarly, miR-184 may participate in BMP signaling pathway by down-regulating GDF3 of module 5 mediated by TFs NANOG. MiR-184 also will participate in signaling pathways such as Endocrine resistance by up-regulating ESR1 of module 1 mediated by STAT5B. In addition, the expression levels of key genes CLU, ESR1, GDF3, and UGT2B15 have been verified by qPCR, consistent with the results predicted previously. In addition, miR-184 expression levels have been verified in human and canine (Fig. 4C).

## Discussion

Hydatidosis is a zoonotic parasitic disease caused by the larva of *Echinococcus*. It not only seriously affects human health, but also causes economic losses in many countries (Longman & Martin, 1987). In addition, alveolar *Echinococcosis* (AE) is the main disease caused by the larval *E.multilocularis* (Gottstein et al., 2017). The infection often involves the liver and may transfer to the lungs and brains (Detry et al., 2018). In recent years, the exploration of echinococcosis has focused on some genes or proteins, as well as related signaling pathways, and some achievements have been made. However, the global regulation of these genes, proteins and signaling pathways in hydatidosis remains unclear. We have synthesized a series of analytical methods to explore the potential molecular mechanism of the occurrence and development of alveolar echinococcosis. Firstly, two groups of dysfunction modules were identified based on protein interaction network and functional enrichment analysis. One is about 16 modules of human-related diseases and the other is about 5 modules of dog-related diseases. Furthermore, we found that TFs as pivot regulators can connect a series of functions and pathways, thus exacerbating the pathological changes of echinococcosis. Based on the results of the enrichment analysis, we found that both human and canine module genes are mainly involved in the “TGF- *β* signaling pathway” and the “BMP signaling pathway”. According to the enrichment analysis, we observed that the physiological process of apoptosis was significantly enriched in seven dysfunction modules in humans and in four dysfunction modules in canine. It happens to confirm that apoptosis is involved in the pathogenesis of *E.multilocularis* (Zhang et al., 2012). Human have 5 dysfunction modules that significantly enrich the regulation of interleukin, and canine have 4 dysfunction modules that significantly enrich the biological process. Samten B et al. also pointed out that the expression levels of interleukin-21 and interleukin-4 in CE patients were significantly higher than those in normal people, which indicated that interleukin was also closely related to the pathogenesis of *E.multilocularis* (Zhang et al., 2015). These functions and pathways involved by module genes have a comprehensive network effect, which comprehensively regulates the physiological disorders caused by *E.multilocularis*.

Secondly, we identified the key genes in these dysfunction modules (including RPS6, RPS3 and RPL5 in humans, and EHMT1 and ACTL9 in canine). Among them, RPS6 phosphorylation has a certain regulatory role in the process of cell cycle. Casein kinase 1 (CK1) dependent phosphorylation of RPS6 promotes the binding of RPS6 to the cap-binding complex. In addition, protein phosphatase 1 (PP1) can antagonize the phosphorylation of RPS6 C terminal and cap binding in intact cells. These findings further deepen our understanding of the regulation of phosphorylation of RPS 6 (Hutchinson et al., 2011; Rosner, Schipany & Hengstschlager, 2013). EHMT1 plays a negative regulatory role in the gene induction pathway mediated by NF-kappa B and interferon I (Ea et al., 2012).

Finally, we identified ncRNA (including CRNDE both in human and canine) and TFs (including NFKB1, TP53, BRCA1 and KLC2) that significantly regulated these dysfunction modules. Statistical analysis showed that long non-coding RNA (lncRNA), mainly CRNDE, played an important regulatory role in the functional dysfunction modules of human and canine. NFKB1 significantly regulates 4 dysfunction modules of human hydatidosis. Related studies have reported that NFKB1 is the core TF regulating hydatidosis, and it may participate in the inflammatory process of hydatidosis (Jiang et al., 2016). Therefore, NFKB1 may play an important regulatory role in the pathogenesis of *E.multilocularis*. In addition, TP53 plays a key role in the regulation of oxidative stress and apoptotic network, so it is considered that TP53 is also involved in the infection of hydatid (Ichimura et al., 2002). The interaction between KLC2 and 14 − 3 − 3 protein depends on the phosphorylation of KLC2, and then mediates the signal transduction pathway (Cheng et al., 2015). These studies indicate that KLC2 plays an important role in the functional pathway of hydatidosis. It is worth noting that the miR-184 of hydatid worms can target multiple module genes through TF and ncRNA, and play multiple regulatory roles. Therefore, we boldly speculate that it is involved in mediating oxidative stress, and that cytotoxic cytokine-induced beta cell dysfunction aggravates the pathological changes after human infection with *E.multilocularis (Barrett et al., 2013)*. In addition, miR-184 may regulate the function of pancreatic beta-cells through glucose metabolism, alter insulin secretion, and thus down-regulate the GDF3 gene, leading to the final infection of hydatidosis in canine (Dalgaard & Eliasson, 2017).

## Conclusions

Based on the results of this study, we have obtained a more comprehensive pathogenic module of *E.multilocularis*. These modules involve many hydatidosis-related genes which been confirmed and candidate factors to be tested, which provide a theoretical basis for further research on hydatidosis. After systematic research, we believe that miR-184 is a key regulatory gene for *E.multilocularis* to promote host disease progression.

##  Supplemental Information

10.7717/peerj.9280/supp-1 Supplemental Information 1 Differential Genes in Dogs First Infected with Hydatidosis

10.7717/peerj.9280/supp-2 Supplemental Information 2 Protein difference quantitative experiment results

10.7717/peerj.9280/supp-3 Supplemental Information 3 GO Function of Human Hydatidosis Function Module

10.7717/peerj.9280/supp-4 Supplemental Information 4 Enrichment results of functional modules of canine echinococcosis

10.7717/peerj.9280/supp-5 Supplemental Information 5 Driving Genes of Human Hydatidosis

10.7717/peerj.9280/supp-6 Supplemental Information 6 ncRNA Driving the Pathogenic Module of Canine Hydatidosis

10.7717/peerj.9280/supp-7 Supplemental Information 7 Pivot prediction of human hydatidosis module based on the regulation of transcription factors to genes

## Additional Information and Declarations

### Data Availability

The following information was supplied regarding data availability: Data is available at Github: https://github.com/111-smx/Sample-data.
